# Unveiling the improvement mechanisms of moist-heat and hot-air treatments on the flavor of *Panax quinquefolius*

**DOI:** 10.1038/s41538-026-00888-3

**Published:** 2026-05-19

**Authors:** Hong-Juan Hou, Wen-Han Pei, Rui-Bo Sun, Chao Wang, Fang-Tong Liu, Tian-Qi Zhang, Gui-Zhong Xin, Cheng Qian, Mu Zhang, Xin-Ru Zhang, Zi-Xuan Ding, Hui Zhang, Tian-Min Wang, Ting-Guo Kang, Hai-Bo Wang, Yue-Hua Chen, Hui-Peng Song

**Affiliations:** 1 Key Laboratory for Identification and Quality Evaluation of Traditional Chinese Medicine of Liaoning Province, Liaoning University of Traditional Chinese Medicine, Dalian, China; 2 The MOE Key Laboratory of Standardization of Chinese Medicines, Shanghai University of Traditional Chinese Medicine, Shanghai, China; 3 College of Pharmacy, Dalian Medical University, Dalian, China; 4 State Key Laboratory of Natural Medicines, China Pharmaceutical University, Nanjing, China; 5 Department of Pathology, School of Basic Medical Sciences, Nanjing Medical University, Nanjing, China; 6 Department of Endocrinology, Sir Run Run Hospital, Nanjing Medical University, Nanjing, China

**Keywords:** Biochemistry, Chemistry, Plant sciences

## Abstract

China-grown American ginseng, namely *Panax quinquefolius* (PQ), faces significant limitations in food industry applications due to its undesirable flavor. Moist-heat and hot-air treatments (MHHA) are one of the main methods for processing PQ. However, its impact on both flavor and functional components of PQ remained unclear. To address this issue, we proposed a three-step strategy termed “HE + H + EH”, comprising the integration of human sensory evaluation and electronic tongue (HE), HPLC‑Q‑TOF‑MS (H), and the integration of electronic nose and HS‑GC‑IMS (EH). Human sensory evaluation and electronic tongue analysis demonstrated that MHHA effectively improved the taste of PQ by significantly reducing its bitterness while enhancing its umami taste. Using HPLC‑Q‑TOF‑MS, we identified 75 compounds in PQ and 90 in processed *Panax quinquefolius* (PPQ). Notably, the content of five bitter malonylginsenosides decreased significantly, which contributed to the reduction in PQ’s bitterness. Meanwhile, five newly generated rare bioactive saponins were detected: vinaginsenoside R_7_, ginsenosides Rk_1_, Rg_5_, Rg_6_, and Rh_4_. Electronic nose and HS‑GC‑IMS analyses revealed that MHHA reduced the levels of undesirable odorants. This study highlighted the role of MHHA in improving the flavor and generating functional ingredients in PQ. These findings provided a solid foundation for the application of PQ in food development.

## Introduction

*Panax quinquefolius* L. (PQ), also known as American ginseng, is an important functional food native to North America. It was successfully introduced and cultivated on a large scale in China in the 1980s^1^. PQ is renowned for its rich bioactive components, with a complex and diverse chemical composition that includes saponins, volatile oils, amino acids, sugars, and polyacetylenic alcohols. Among these, saponins are the primary constituents^2^. PQ possesses both medicinal and dietetic value and is widely used in the processing of various health foods^3^. However, the natural earthy flavor and bitterness of PQ severely limit its development and application in the food industry. Furthermore, traditional processing methods, such as sun-dried PQ, present several drawbacks, including prolonged drying cycles, flavor defects, and poor process controllability. Ultimately, these issues lead to a decline in product quality and a waste of resources. In recent years, combined processing technologies have become a research focus for improving PQ quality through multi-stage synergistic effects. Moist-heat processing (MH) employs high temperature and humidity to soften the tissue structure and facilitate the formation of active components. Hot-air drying (HA) uses forced convection to accelerate moisture transfer, thus helping to enhance the sensory quality of the product. Studies have shown that MH can significantly increase the contents of several ginseng saponins^4^. Additionally, HA can markedly shorten the drying time^5^. Both techniques show significant effects when applied individually. However, the combined impact of moist-heat and hot-air treatments (MHHA) on the flavor and composition of PQ remains unclear.

In food research, the innovative combination of multiple technologies enables efficient and precise analysis of food flavor and composition. In electronic sensory evaluation, electronic tongue and electronic nose play indispensable roles. While conventional human sensory evaluation captures people’s real opinions about food, the results are not always reliable. They can be skewed by an evaluator’s personal experience, the conditions of the test, and inconsistent rating scales. Consequently, the evaluation outcomes exhibit a degree of inherent subjectivity and variability. In contrast, electronic sensory technologies effectively compensate for these shortcomings with their objectivity and reproducibility. For example, changes in the sensory characteristics of strawberries during five‑day storage were evaluated by utilizing the relationship between human and electronic senses^6^. In another study, a systematic evaluation of Italian wines was conducted based on a combination of electronic tongue and human sensory perception^7^. Among volatile compound detection technologies, HS-GC-IMS is characterized by rapid response, high sensitivity, simple operation, and cost-effectiveness^8^. For instance, volatile flavor compounds in five dried fish samples were successfully distinguished using HS-GC-IMS^9^. By employing HS-GC-IMS, 35, 29, 8, and 12 key volatile components were respectively identified from four Baijiu samples^10^. The high sensitivity, high resolution, and strong qualitative and structural analysis capabilities of HPLC-Q-TOF-MS make it an ideal tool for the comprehensive analysis of non-volatile food components^11^. For example, 117 compounds were identified from *Zanthoxylum nitidum* using HPLC-Q-TOF-MS^12^. A total of 103 compounds were identified and categorized from *Magnolia officinalis* using HPLC-Q-TOF-MS^13^. The integrated application of these technologies in flavor evaluation has become an inevitable trend in the food industry.

This study aimed to elucidate the enhancement mechanisms of MHHA on the flavor and functional components of China-grown PQ, thereby providing a theoretical basis for the development and quality enhancement of PQ products. To this end, we integrated five techniques, including human sensory evaluation (H), electronic tongue (E), HPLC-Q-TOF-MS (H), electronic nose (E), and HS-GC-IMS (H), into a three-step strategy termed “HE + H + EH”. First, human sensory evaluation and electronic tongue (HE) were integrated to comprehensively and accurately characterize the taste enhancement of PQ by MHHA. Second, HPLC-Q-TOF-MS (H) was employed to investigate the alterations in taste and functional components of PQ induced by MHHA. Third, electronic nose and HS-GC-IMS (EH) were combined to comprehensively analyze the changes in odor components of PQ resulting from MHHA. The integration of the HE, H, and EH steps enabled a systematic elucidation of the mechanism by which MHHA improves the flavor of PQ. This established a crucial foundation for the further development of China-grown American ginseng in the food industry.

## Results

### Three-step “HE + H + EH” strategy

**Fig. 1:**
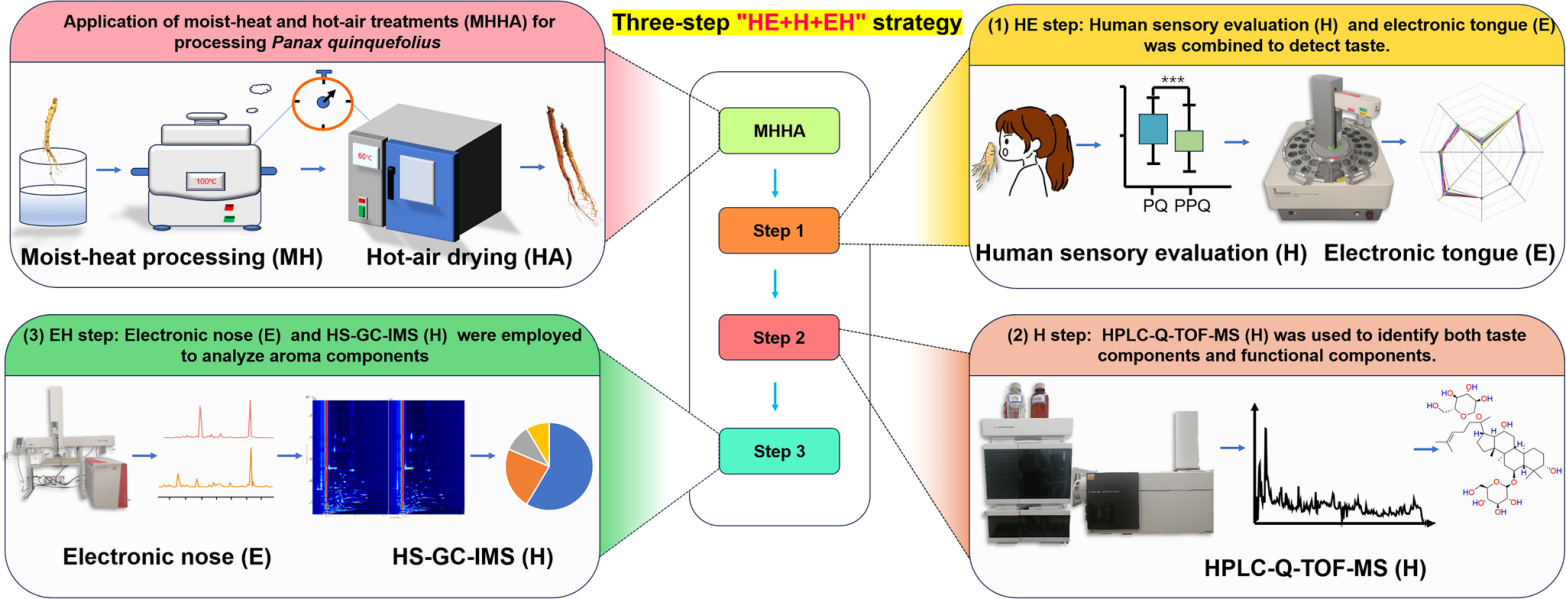
Flowchart of the research strategy for PQ and PPQ. The workflow begins with the moist-heat and hot-air treatments. Step 1: Integration of human sensory evaluation and the electronic tongue for the analysis of taste characteristics; Step 2: Identification of potential taste and functional components using HPLC-Q-TOF-MS; Step 3: Combination of the electronic nose and HS-GC-IMS for the analysis of volatile aroma compounds.

To systematically decipher the multifaceted effects of MHHA treatment, we designed an integrated analytical strategy that progressed from sensory perception to molecular characterization (as illustrated in Fig. 1). This “HE + H + EH” strategy was intentionally structured to establish causal links between sensory attributes and their underlying chemical constituents. First, the combined human sensory evaluation and electronic tongue (HE) analysis quantified the holistic taste profile, thereby defining the key sensory outcomes produced by the treatment. Subsequently, HPLC-Q-TOF-MS (H) was employed to target the non-volatile compound pool, identifying the specific taste-active and functional components responsible for the sensory changes measured in the first step. Finally, the electronic nose coupled with HS-GC-IMS (EH) mapped the volatile composition, thereby completing a comprehensive flavor assessment by linking aroma changes to the process. Therefore, the “HE + H + EH” strategy provided a clear roadmap for this investigation, constructing an evidence chain from sensory perception to chemical causation.

### The enhancing effect of MHHA on the flavor of PQ

**Fig. 2:**
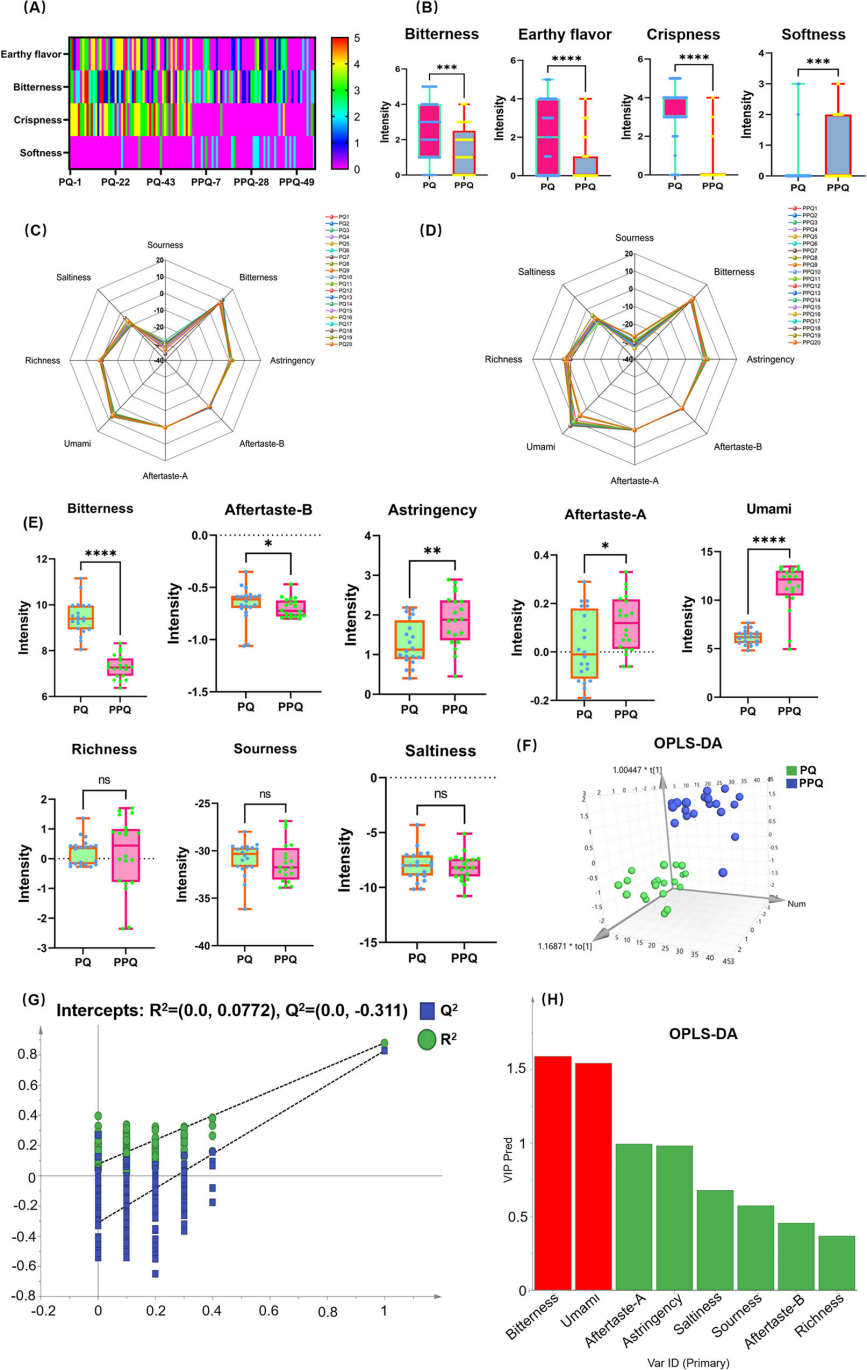
Differential flavor analysis of PQ and PPQ based on human sensory evaluation and electronic tongue. **A** Heatmap of taste attribute clustering based on human sensory evaluation scores of PQ and PPQ. Color scale: blue to red represents low to high intensity. **B** Box plots of earthy flavor, bitterness, softness, and crispness distribution in PQ and PPQ. **C** Radar chart of PQ based on electronic tongue data. **D** Radar chart of PPQ based on electronic tongue data. Aftertaste-B refers to aftertaste-bitterness, while Aftertaste-A refers to aftertaste-astringency. **E** Box plots of quantitative scoring differences between PQ and PPQ across eight taste sensors. Statistical significance indicated as * *p* ≤ 0.05, ** *p* ≤ 0.01, *** *p* ≤ 0.001, **** *p* ≤ 0.0001 and ns for not significant. **F** OPLS‑DA plot of PQ (green) and PPQ (blue) samples based on electronic tongue data. **G** Permutation test (*n* = 200) for validation of the OPLS-DA model. **H** VIP score plot of taste attributes for discrimination between PQ and PPQ. Red represents taste attributes with VIP > 1.

The results of human sensory evaluation are shown in Fig. 2A, B. PPQ showed reductions in earthy flavor, bitterness, and crispness, while its softness increased significantly. It should be noted that earthy flavor is essentially a retronasal olfactory sensation, which is fundamentally different from the basic taste attributes^14^. The retronasal olfaction does not directly alter the recognition of basic tastes, but the inherent earthy flavor of PQ can synergize with bitterness to further intensify negative sensory perception. The decrease in crispness was closely related to the reduction in moisture content of PQ from 63.08 to 7.11%. Compared with traditional sun-drying which takes approximately 14.7 days, the processing time of MHHA is 13 hours, representing a reduction of more than 90%^5^. The improved softness may result from the moist-heat treatment disrupting rigid cell walls and reducing tissue strength, combined with the hot-air drying preventing shrinkage and preserving the loose structure^15,16^. This resulted from the synergistic effect of MHHA. The treatment improved the softness of PQ while reducing its unpleasant taste, thus yielding a softer and more palatable mouthfeel. Subsequently, the electronic tongue analysis quantified eight sensory dimensions: sourness, bitterness, saltiness, astringency, umami, richness, and the aftertastes of bitterness and astringency. Radar charts for PQ and PPQ are shown in Fig. 2C, D, respectively. The quantitative results for the eight taste attributes are presented in Fig. 2E. Compared with PQ, PPQ showed reduced bitterness (decreasing from 9.4375 to 7.2990) and significantly enhanced umami (increasing from 6.1710 to 11.3065). By combining human sensory evaluation with electronic tongue analysis, it was demonstrated that MHHA improved the taste quality of PQ.

To further validate these findings, orthogonal partial least squares discriminant analysis (OPLS-DA) was applied to visualize and classify the data. As shown in Fig. 2F, the OPLS-DA model effectively discriminated between PQ and PPQ. The model showed high stability (R²Y = 0.881) and predictive capability (Q² = 0.829), confirming the reliability of the analysis. Permutation tests further validated the model’s robustness, yielding R²Y and Q²Y intercepts of 0.0772 and –0.311, respectively, both of which were well below the critical thresholds of 0.40 and 0.05 (Fig. 2G)^17^. The variable importance in projection (VIP) analysis revealed that bitterness and umami (VIP > 1) were the taste indicators that significantly contributed to distinguishing PQ from PPQ (Fig. 2H). This provided additional evidence that MHHA had a significant impact on the taste of PQ. In brief, using human sensory evaluation and an electronic tongue improved the taste of PQ by reducing bitterness and earthy flavor, while enhancing umami and softness.

### Impact of MHHA on the composition of PQ

**Fig. 3:**
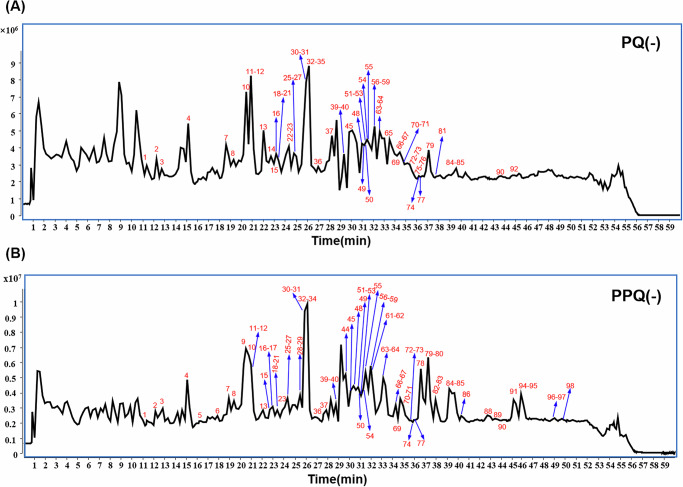
Comparison of components of PQand PPQ by HPLC-Q-TOF-MS. **A** Total ion chromatogram of PQ in negative ion mode. **B** Total ion chromatogram of PPQ in negative ion mode. Peak numbers correspond to the compounds listed in Table 1. Identical chromatographic conditions were applied to both samples to facilitate direct constituent comparison.

HPLC-Q-TOF-MS was used for qualitative analysis and comparative analysis of component levels in 40 batches each of PQ and PPQ. The total ion chromatograms (TICs) of PQ and PPQ in negative ion mode are shown in Fig. 3A, B, respectively. The positive ion mode TICs are shown in Fig. S1A and Fig. S1B. The identification was based on exact relative molecular mass, retention time, mass spectrometric fragments, literature data, and comparison with reference substances. In addition, published data and specialized databases were also employed to identify ginsenosides^18,19^. As a result, a total of 98 compounds were identified from PQ and PPQ (Table 1). Of these, 75 and 90 compounds were specifically identified from PQ and PPQ, respectively. Sixty-seven saponins were common components shared by both PQ and PPQ. PQ contained 8 unique constituents, while PPQ contained 23 unique constituents.

**Table 1:** Chemical constituents identified in PQ and PPQ via HPLC-Q-TOF-MS

No.	t_R_ (min)	m/z (Error, ppm)	Formula	Fragment ions (m/z)	Identification	Distribution
1	11.418	861.4864 (-1.32)^+HCOO^	C_42_H_72_O_15_	391.2839、491.3698、553.3395、653.4286、655.3815、815.4802	Momorcharassikde A	PQ、PPQ
2	12.334	1007.5436 (-0.38)^+HCOO^	C_48_H_82_O_19_	637.4221	Notoginsenoside R_3_	PQ、PPQ
3	12.535	1007.5433 (-0.07)^+HCOO^	C_48_H_82_O_19_	637.4273	Ginsenoside Re_1_	PQ、PPQ
4	14.768	861.4871 (-2.17)^+HCOO^	C_42_H_72_O_15_	391.2851、491.3724、653.4281、655.4353、815.4706	Majonoside R_1_	PQ、PPQ
5	16.749	977.5332 (-0.57)^+HCOO^	C_47_H_80_O_18_	931.5263	Ginsenoside Re_4_	PPQ
6	18.479	977.5329 (-0.25)^+HCOO^	C_47_H_80_O_18_	475.3794、637.4328、931.5272	Quinquenoside L_17_	PPQ
7	19.071	1007.5429 (-0.35)^+HCOO^	C_48_H_82_O_19_	637.4331、799.4692	Ginsenoside Re_2_	PQ、PPQ
8	19.328	977.5339 (-1.32)^+HCOO^	C_47_H_80_O_18_	475.3775、637.4323、751.4701、769.4755、799.4853、931.5279	Notoginsenoside R_1_	PQ、PPQ
9	20.091	991.5500 (-1.78)^+HCOO^	C_48_H_82_O_18_	475.3827、619.4249、637.4325、765.4797、783.4933、799.4850、945.5431	Ginsenoside Re*	PPQ
10	20.064	1025.5576 (-3.88)^+HCOO^	C_48_H_84_O_20_	655.4438、817.4960	Vinaginsenoside R_13_	PQ、PPQ
11	20.343	845.4917 (-1.61)^+HCOO^	C_42_H_72_O_14_	799.4263、637.4303、619.4169、475.3765	Ginsenoside Rf	PQ、PPQ
12	20.505	845.4964 (-7.48)^+HCOO^	C_42_H_72_O_14_	475.3793、637.4318	Ginsenoside Ia	PQ、PPQ
13	21.046	845.4899 (0.64)^+HCOO^	C_42_H_72_O_14_	475.3781、799.4851	Ginsenoside Rg_7_	PQ、PPQ
14	22.074	885.4853 (0.03)^-H^	C_45_H_74_O_17_	475.3805、781.4743	Ginsenoside mRf	PQ
15	22.532	1031.5446 (-1.32)^-H^	C_51_H_84_O_21_	637.4341、783.4857、945.5421、987.5527	Malonylginsenoside Re	PQ、PPQ
16	22.682	1139.5860 (-0.46)^+HCOO^	C_53_H_90_O_23_	1093.5803、781.4745	Floranotoginsenoside A	PQ、PPQ
17	22.693	1139.5850 (0.45)^+HCOO^	C_53_H_90_O_23_	781.4732、1093.5807	Yesanchinoside H	PPQ
18	22.715	1139.5859 (-0.37)^+HCOO^	C_53_H_90_O_23_	619.4169、781.4740	Floranotoginsenoside D	PQ、PPQ
19	22.733	1139.5866 (-1.01)^+HCOO^	C_53_H_90_O_23_	475.3800、637.4314、781.4749、961.5351、1093.5798	Gypenoside LXIX	PQ、PPQ
20	22.828	1139.5870 (-1.38)^+HCOO^	C_53_H_90_O_23_	637.4267、1093.5795	Gypenoside LXXI	PQ、PPQ
21	22.861	961.5378 (-0.05)^+HCOO^	C_47_H_80_O_17_	915.5330	Vinaginsenoside R_16_	PQ、PPQ
22	23.564	885.4864 (-1.21)^-H^	C_45_H_74_O_17_	475.3754、619.4158	Malonylginsenoside Rg_1_	PQ
23	24.005	815.4798 (0.06)^+HCOO^	C_41_H_70_O_13_	769.4263、637.4303、619.4169、475.3765	Notoginsenoside R_2_	PQ、PPQ
24	24.077	793.4696 (1.64)^+Na^	C_41_H_70_O_13_	793.4696	Ginsenoside F_5_	PQ、PPQ
25	24.107	961.5391 (-1.47)^+HCOO^	C_47_H_80_O_17_	459.3506、783.4882、915.5314	Vinaginsenoside R_17_	PQ、PPQ
26	24.134	815.4832 (-4.35)^+HCOO^	C_41_H_70_O_13_	769.4710、637.4325、619.4142、475.3782	Notoginsenoside F_3_	PQ、PPQ
27	24.151	815.4810 (-1.41)^-H^	C_53_H_90_O_24_	475.3802、637.4327	Floralginsenoside C	PQ、PPQ
28	24.642	887.5024 (-1.69)^+HCOO^	C_44_H_74_O_15_	619.4246、475.3778、781.4760、841.4939	Acetylginsenoside Rg_1_	PPQ
29	24.654	887.5019 (-1.10)^+HCOO^	C_44_H_74_O_15_	841.4889	Yesanchinoside D (6′-O-acetyl-ginsenoside Rg_1_)	PPQ
30	24.793	1033.5589 (-0.02)^+HCOO^	C_50_H_84_O_19_	621.4143、765.4804、783.4912、927.5280、945.5429	Quinquenoside III	PQ、PPQ
31	24.845	1033.5609 (-2.04)^+HCOO^	C_50_H_84_O_19_	475.3810、637.4327、799.4877	6″-O-acetylginsenoside Re	PQ、PPQ
32	25.022	1007.5449 (-1.73)^+HCOO^	C_48_H_82_O_19_	637.4313、799.4858	Ginsenoside Re_3_	PQ、PPQ
33	25.379	831.4746 (0.20)^+HCOO^	C_41_H_70_O_14_	653.4277	Vinaginsenoside R_11_	PQ、PPQ
34	25.412	831.4742 (0.71)^+HCOO^	C_41_H_70_O_14_	653.4257、785.4675	Majonoside R_2_	PQ、PPQ
35	25.737	699.4340 (-2.29)^+HCOO^	C_36_H_62_O_10_	491.3778	Pseudoginsenoside Rt	PQ
36	26.473	845.4922 (-2.24)^+HCOO^	C_42_H_72_O_14_	491.3743、637.4363	Majoroside F_3_	PQ、PPQ
37	28.027	815.4809 (-1.37)^+HCOO^	C_41_H_70_O_13_	769.4695、637.4313、475.3791	Notoginsenoside F_5_	PQ、PPQ
38	28.264	793.4705 (0.47)^+Na^	C_41_H_70_O_13_	793.4695、335.0951	Ginsenoside F_3_	PQ、PPQ
39	29.366	829.4963 (-1.03)^+HCOO^	C_42_H_72_O_13_	475.3798、619.4229、637.4326	20*(S)*-Ginsenoside Rg_2_*	PQ、PPQ
40	29.462	1193.5987 (-2.21)^-H^	C_57_H_94_O_26_	459.3874、621.4375、765.4775、783.4895、927.5297、945.5435、1089.5847、1107.5955、1149.6071	Malonylginsenoside Rb_1_	PQ、PPQ
41	29.626	1131.5919 (0.23)^+Na^	C_54_H_92_O_23_	1131.5892、365.1041	Ginsenoside Rb_1_*	PQ、PPQ
42	29.666	1101.5809 (0.64)^+Na^	C_53_H_90_O_22_	407.3681、767.4951	Ginsenoside Rb_2_*	PQ、PPQ
43	29.838	1101.5810 (0.55)^+Na^	C_53_H_90_O_22_	789.4761	Ginsenoside Rc*	PQ、PPQ
44	30.053	1123.5918 (-1.13)^+HCOO^	C_53_H_90_O_22_	945.5374	Vinaginsenoside R_7_	PPQ
45	30.265	1163.5871 (-1.38)^-H^	C_56_H_92_O_25_	459.3855、621.4371、783.4899、945.5446、1077.5855	Malonylvinaginsenoside R_7_	PQ、PPQ
46	30.731	1101.5814 (0.18)^+Na^	C_53_H_90_O_22_	789.4781	Ginsenoside Rb_3_*	PQ、PPQ
47	30.811	979.4857 (1.67)^+Na^	C_48_H_76_O_19_	379.0813、641.3976、979.4855	Ginsenoside Ro	PQ、PPQ
48	31.031	955.4903 (0.53)^-H^	C_48_H_76_O_19_	793.4387	Hemsgiganoside B	PQ、PPQ
49	31.147	1163.5849 (0.51)^-H^	C_56_H_92_O_25_	621.4373、783.4899、1077.5853、1119.5944	Malonylginsenoside Rc	PQ、PPQ
50	31.260	1163.5834 (1.80)^-H^	C_56_H_92_O_25_	459.3820、621.4326、783.4898、945.5425、1077.5850	Malonylginsenoside Rb_3_	PQ、PPQ
51	31.343	955.4917 (-0.94)^-H^	C_48_H_76_O_19_	793.4364	Tuberoside A	PQ、PPQ
52	31.443	1087.5374 (-3.98)^-H^	C_53_H_84_O_23_	955.4902	OA-28-Glc-Glc-3-GlurA-Xyl	PQ、PPQ
53	31.522	1163.5855 (-0.01)^-H^	C_56_H_92_O_25_	459.3838、621.4378、765.4820、783.4925、915.5330、945.5425、1059.5745、1077.5852、1119.5910	Malonylginsenoside Rb_2_	PQ、PPQ
54	31.606	1195.6131 (-1.30)^+HCOO^	C_56_H_94_O_24_	459.3791、621.4360、765.4809、783.4886、927.5330、945.5417、1089.5857、1107.5961、1149.6045	Acetylginsenoside Rb_1_	PQ、PPQ
55	31.690	925.4797 (0.58)^-H^	C_47_H_74_O_18_	569.3858	Chikusetsusaponin Ib	PQ、PPQ
56	31.718	925.4823 (-2.22)^-H^	C_47_H_74_O_18_	569.3858、613.3754、763.4294	Pseudoginsenoside RT_1_	PQ、PPQ
57	31.734	925.4790 (1.34)^-H^	C_47_H_74_O_18_	613.3746、569.3857	Chikusetsusaponin IV	PQ、PPQ
58	31.751	925.4802 (0.04)^-H^	C_47_H_74_O_18_	455.3518、497.3628、523.3777、569.3854、583.4036、587.3964、613.3751、719.4379、763.4277	Stipuleanoside R_1_	PQ、PPQ
59	31.755	1149.6075 (-1.11)^-H^	C_56_H_94_O_24_	621.4383、765.4791、783.4908、927.5124、945.5465、1089.5862、1107.5966	Quinquenoside R_1_	PQ、PPQ
60	32.205	969.5385 (0.88)^+Na^	C_48_H_82_O_18_	789.4769、365.1076	Ginsenoside Rd*	PQ、PPQ
61	32.460	1165.6016 (-0.41)^+HCOO^	C_55_H_92_O_23_	621.4355、783.4945、945.5440、1059.5752、1077.5830	Ginsenoside Rs_1_	PPQ
62	32.571	1165.6017 (-0.50)^+HCOO^	C_55_H_92_O_23_	459.3823、621.4375、783.4913、945.5420、1059.5734、1077.5827	Ginsenoside Rs_2_	PPQ
63	32.912	793.4392 (-1.54)^-H^	C_42_H_66_O_14_	455.3529、587.3950、613.3755	Zingibroside R_1_	PQ、PPQ
64	32.929	793.4399 (-2.42)^-H^	C_42_H_66_O_14_	455.3538、497.3657、569.3861、587.3962、613.3761、631.3858、673.3945	Spinasaponin A	PQ、PPQ
65	33.961	1117.5439 (-0.24)^-H^	C_54_H_86_O_24_	987.5533、945.5419、783.4927、459.3845	Dimalonylginsenoside Rd	PQ
66	34.208	961.5394 (-1.80)^+HCOO^	C_47_H_80_O_17_	621.4376、459.3818	Notoginsenoside Fd	PQ、PPQ
67	34.236	961.5405 (-3.00)^+HCOO^	C_47_H_80_O_17_	459.3838、621.4369	Notoginsenoside Fe	PQ、PPQ
68	34.245	939.5287 (0.08)^+Na^	C_47_H_80_O_17_	335.0938、939.5286	Ginsenoside Rd_2_	PQ、PPQ
69	34.252	961.5370 (0.82)^+HCOO^	C_47_H_80_O_17_	621.4377、783.4874、915.5279	Compound O	PQ、PPQ
70	34.693	961.5381 (-0.38)^+HCOO^	C_47_H_80_O_17_	915.5323、783.4901、621.4376、459.3818	Gynosaponin I	PQ、PPQ
71	34.710	961.5390 (-1.36)^+HCOO^	C_47_H_80_O_17_	915.5327、783.4903、621.4384、459.3839	Vinaginsenoside R_18_	PQ、PPQ
72	35.008	1033.5591 (-0.22)^+HCOO^	C_50_H_84_O_19_	459.3842、603.4288、621.4275、765.4734、783.4828、927.5298、945.5447	Pseudoginsenoside Rc_1_	PQ、PPQ
73	35.088	1033.5583 (0.59)^+HCOO^	C_50_H_84_O_19_	783.4828、161.0463	Quinquefolium III	PQ、PPQ
74	35.352	961.5361 (1.80)^+HCOO^	C_47_H_80_O_17_	621.4387、783.4878、915.5327	Gypenoside IX	PQ、PPQ
75	35.380	1001.5342 (-1.53)^-H^	C_50_H_82_O_20_	915.5324、621.4368、459.3831	Malonylvinaginsenoside R_18_	PQ
76	35.413	1001.5329 (-0.23)^-H^	C_50_H_82_O_20_	459.3847、621.4378、783.4913、915.5305	Malonylnotoginsenoside Fe	PQ
77	35.496	961.5399 (-2.34)^+HCOO^	C_47_H_80_O_17_	459.3846、621.4367	Notoginsenoside Ft_1_	PQ、PPQ
78	36.827	811.4867 (-2.31)^+HCOO^	C_42_H_70_O_12_	161.0464	Ginsenoside Rg_4_	PPQ
79	37.134	829.4967 (-1.54)^+HCOO^	C_42_H_72_O_13_	113.0246、143.0450、161.0456、621.4365、783.4903	Ginsenoside F_2_*	PQ、PPQ
80	37.346	811.4849 (0.04)^+HCOO^	C_42_H_70_O_12_	101.0251、113.0250、125.0251、143.0352、161.0462、205.0725、457.3671、601.4065、619.4225、765.4775	Ginsenoside Rg_6_	PPQ
81	37.446	1059.5748 (-0.26)^+HCOO^	C_52_H_86_O_19_	161.0431、783.4905	Quinquefolium I	PQ
82	37.972	665.4290 (-3.19)^+HCOO^	C_36_H_60_O_8_	113.0253、161.0446	Ginsenoside Rh_4_	PPQ
83	38.050	665.4286 (-2.54)^+HCOO^	C_36_H_60_O_8_	161.0446	Ginsenoside Rk_3_	PPQ
84	39.473	829.4955 (-0.01)^+HCOO^	C_42_H_72_O_13_	101.0243、113.0244、143.0359、161.0458、459.3850、621.4370、	20*(S)*-Ginsenoside Rg_3_*	PQ、PPQ
85	39.941	829.4961 (-0.77)^+HCOO^	C_42_H_72_O_13_	101.0253、113.0246、143.0357、161.0459、459.3855、621.4387、783.4907	20*(R)*-Ginsenoside Rg_3_*	PQ、PPQ
86	40.527	871.5059 (0.19)^+HCOO^	C_44_H_74_O_14_	783.4860、621.4357、459.3794	Ginsenoside Rs_3_	PPQ
87	40.603	777.4749 (1.39)^+Na^	C_41_H_70_O_12_	335.0968、777.4755	Ginsenoside C-Y	PPQ
88	42.083	763.4282 (-1.03)^-H^	C_41_H_64_O_13_	569.3825	Pseudoginsenoside Rp_1_	PPQ
89	42.875	871.5076 (-1.86)^+HCOO^	C_44_H_74_O_14_	459.3852、621.4376、783.4919	Acetylginsenoside Rg_3_	PPQ
90	43.572	631.3862 (-1.65)^-H^	C_36_H_56_O_9_	631.3858、455.3533	Calenduloside E	PQ、PPQ
91	44.132	667.4434 (-1.17)^+HCOO^	C_36_H_62_O_8_	459.3874	Compound K	PPQ
92	44.834	723.3809 (-0.06)^+HCOO^	C_33_H_58_O_14_	415.1484、397.1347、379.1245、305.0898、279.2340、235.0812、179.0553、125.0238	Gingerglycolipid B (non-saponin)	PQ
93	45.199	789.4754 (0.72)^+Na^	C_42_H_70_O_12_	365.1018、789.4749	Ginsenoside Rk_1_	PQ、PPQ
94	45.388	811.4862 (-1.66)^+HCOO^	C_42_H_70_O_12_	101.0251、113.0248、125.0253、143.0356、161.0456	Ginsenoside F_4_	PPQ
95	45.549	811.4864 (-1.92)^+HCOO^	C_42_H_70_O_12_	161.0450、765.4796	Ginsenoside Rg_5_	PPQ
96	48.816	853.4958 (-0.38)^+HCOO^	C_44_H_72_O_13_	441.0785、603.4286、747.4677、765.4780、807.4908	Acetylginsenoside Rg_5_	PPQ
97	48.849	853.4986 (-3.84)^+HCOO^	C_44_H_72_O_13_	603.4264、765.4802	Ginsenoside Rs_4_	PPQ
98	49.062	853.4991 (-4.46)^+HCOO^	C_44_H_72_O_13_	441.3752、603.4284	Ginsenoside Rs_5_	PPQ

+ Na: [M+Na]^+^; -H: [M-H]^-^; + HCOO: [M + HCOO]^-^

* The compounds were identified by comparing with reference substances.

**Fig. 4:**
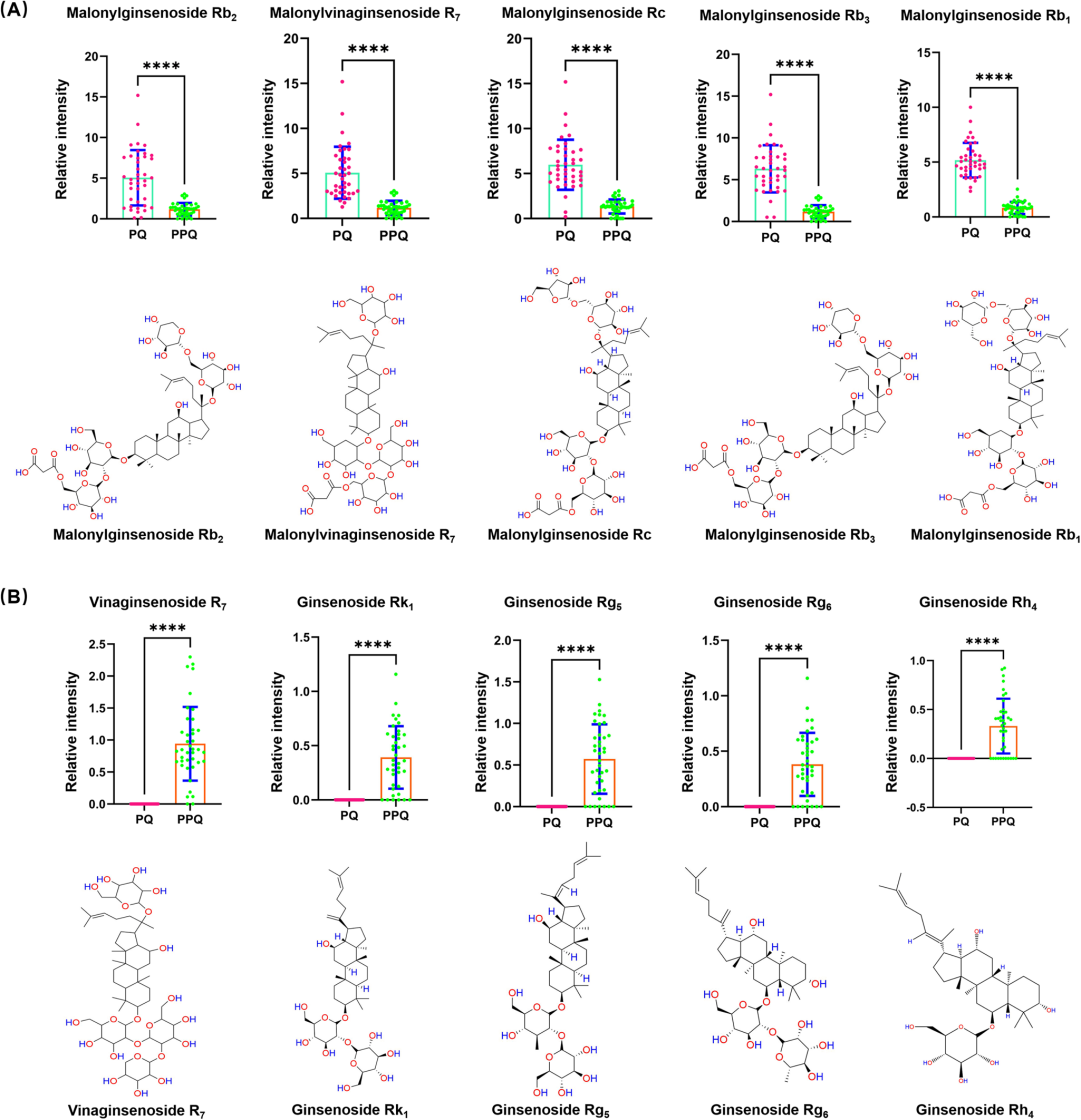
Relative content changes and chemical structural characteristics of ten differential saponins in PQ and PPQ. **A** Significant decrease in five malonylginsenosides in the PPQ group. **B** Significant increase in five newly formed saponins in the PPQ group. **** *p* ≤ 0.0001.

After MHHA, the relative content of five malonylginsenosides in PQ was reduced by more than 50%. These included malonylginsenoside Rb_1_ (by 84.21%), Rb_3_ (by 81.39%), Rc (by 77.65%), Rb_2_ (by 76.78%), and malonylvinaginsenoside R_7_ (by 76.89%). Their content changes and chemical structures are shown in Fig. 4A. Thus, malonylginsenosides exhibited high polarity and strong hydrophilicity due to the presence of carboxyl groups^20^. Previous literature has indicated that this type of saponin is thermally unstable and readily undergoes hydrolysis, losing the malonyl group to yield neutral saponins^21^. This observation likely accounted for the decrease in content of these saponins. Additional literature indicated that the carboxyl group in the malonyl moiety could interact with positively charged regions of bitter taste receptors, thereby contributing to the bitterness^22^. Therefore, the reduction in bitterness in PPQ may be related to the decreased content of malonylginsenosides. Furthermore, the newly generated neutral saponins had reduced hydrophilicity. They were thus more readily absorbed by the body and could have had higher bioavailability. These results indicated that MHHA not only reduced the content of bitter compounds but also promoted the formation of saponins with improved bioavailability.

Five newly transformed compounds were detected in PPQ, including vinaginsenoside R_7_, ginsenoside Rk_1_, ginsenoside Rg_5_, ginsenoside Rg_6_, and ginsenoside Rh_4_. Their content changes and chemical structures are presented in Fig. 4B. Notably, vinaginsenoside R_7_ was the product of malonylvinaginsenoside R_7_ after the removal of the malonyl group. This further demonstrated that malonyl saponins were unstable at high temperatures. In addition, the other four ginsenosides were classified as rare ginsenosides. These newly generated saponins were shown to exert beneficial effects on the human body. For instance, vinaginsenoside R_7_ exerted anti-inflammatory and antioxidant effects through the inhibition of the NF-κB transcription factor activity^23^. Ginsenoside Rk_1_ improved endothelial dysfunction in diabetic vasculature via activation of the PPARγ/eNOS pathway^24^. It promoted endogenous apoptosis in cancer cells through targeted regulation of key genes associated with liver cancer^25^. Ginsenoside Rg_5_ exerted sedative-hypnotic effects by influencing the GABAergic and serotonergic nervous systems^26^. Ginsenoside Rg_6_ inhibited the proliferation of human lymphoma JK cells by influencing mitochondrial dysfunction^27^. Ginsenoside Rh_4_ exerted anti-metastatic effects on gastric cancer by inhibiting the SIX1‑TGF‑β/Smad2/3 signaling axis^28^.

OPLS-DA was performed based on the ratios of the peak areas of the compounds to that of the internal standard in 40 batches of PQ and 40 batches of PPQ samples (Fig. S2A). The samples from the two groups were completely separated. The values of R²X (0.734), R²Y (0.869), and Q² (0.827) demonstrated the validity of the model. Figure S2B shows the results of the permutation test. The R²Y-intercept (0.0162) and Q²-intercept (-0.156) were both below their respective thresholds of 0.40 and 0.05. This result further verified the reliability of the model. Using a VIP > 1 threshold, malonylginsenosides Rb_1_, Rb_3_, Rc, and vinaginsenoside R_7_ were identified as the major differential markers between PQ and PPQ (Fig. S2C). These compounds were all malonylginsenosides or their degradation products, suggesting that MHHA exerted a significant influence on this class of saponins. This series of component transformations may be mainly driven by the MH step of MHHA. The high temperature and humidity environment likely disrupts the cell wall structure of PQ, triggering the hydrolysis of thermolabile malonylginsenosides. Meanwhile, the high-temperature condition may induce the structural rearrangement of ginsenosides, which could be the key reason for the generation of rare bioactive saponins^29^.

### Impact of MHHA on the volatile compounds of PQ

**Fig. 5:**
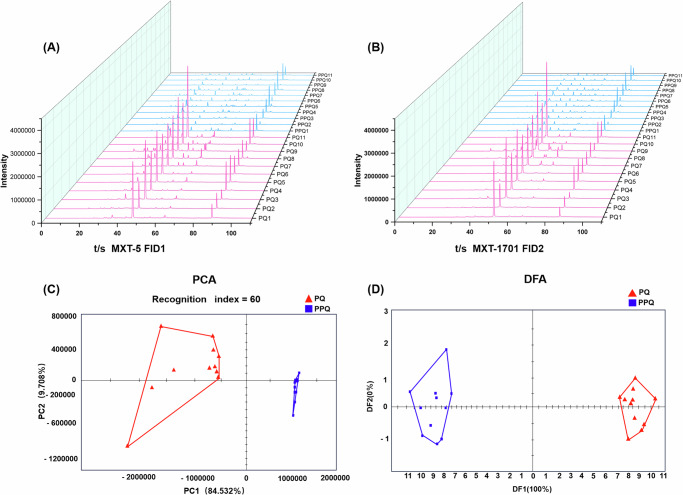
Comparative analysis of PQ and PPQ based on electronic nose and multivariate statistical analysis. **A** Odor profiles of PQ and PPQ on the MXT-5 column. **B** Odor profiles of PQ and PPQ on the MXT-1701 column. **C** PCA score plot of PQ and PPQ samples. **D** DFA score plot of PQ and PPQ samples.

To investigate the impact of MHHA on the volatile components of PQ, an electronic nose was employed to analyze the volatile profile of PQ and PPQ. Fig. 5A presents the comparative results of PQ and PPQ on the MXT-5 column. Similarly, Fig. 5B presents their comparative results on the MXT-1701 column. Evidently, distinct differences were observed between the odor fingerprints of PQ and PPQ. Furthermore, the principal component analysis (PCA) result demonstrated a complete separation between the 11 PQ and 11 PPQ samples (Fig. 5C). The recognition index of PCA reached 60, indicating significant intergroup differences. The contribution rates of PC1 and PC2 were as high as 94.24%. Discriminant factor analysis (DFA) results showed that PQ and PPQ were completely separated, with a cumulative contribution rate of 100% from DF1 and DF2 (Fig. 5D). This further confirmed the odor characteristic differences between PQ and PPQ. The volatile compounds of PQ and PPQ were identified using the AroChemBase database within the electronic nose in combination with Kovats retention indices. With a correlation index greater than 50 as the criterion, 14 components were identified from PQ (Table 2), and 3 components were identified from PPQ (Table 3). Based on the data, the number of some undesirable odor compounds in PPQ was observed to decrease. These substances, including beta-damascenone, 2-methylpropanal, butanoic acid, pentanoic acid, and decyl acetate, were characterized by burnt, spicy, and putrid odors. Concurrently, several pleasant aroma compounds increased in PPQ, such as 3-methylbutanal, methyl isobutyrate, and alpha-pinene. These compounds displayed a spectrum of pleasant aromas, encompassing sweet, fruity, malty, and cheesy notes.

**Table 2:** Volatile compounds in PQ were identified using an electronic nose

No.	RI (MXT-5)	RI (MXT-1701)	Molecular formula	Possible volatile compounds	Index of correlation	Sensory descriptions
1	938	934	C_10_H_16_	alpha-Pinene	62.46	camphor, orange, earthy, fresh, fruity, freshly cut grass, lime flavor, pine, sweet, terpenic, turpentine, woody
2	1381	1500	C_13_H_18_O	beta-Damascenone	69.65	apple, rose apple, canned peach, fruity, honey, rosy, sweet, tobacco
3	534	616	C_4_H_8_O	2-Methylpropanal	63.61	aldehydic, baked potato, burnt, floral, fresh, fruity, freshly cut grass, malty, spicy, lavender, citrus, neroli
4	818	934	C_7_H_16_O	2,3-Dimethyl-1-pentanol	68.48	**/**
5	818	972	C_4_H_8_O_2_	Butanoic acid	79.74	buttery, cheesy, penetrating, rotten, sharp, sweet
6	714	848	C_4_H_8_O_2_	Acetoin	82.66	buttery, coffee, creamy, dairy, fatty, milky, sweet, woody
7	908	1096	C_5_H_10_O_2_	Pentanoic acid	63.99	acidic, beefy, cheesy, penetrating, spicy, putrid, rotten, sour, sweet
8	908	1035	C_4_H_4_O_2_	2-(5H)-Furanone	95.70	buttery
9	938	1052	C_7_H_12_O	3-Hepten-2-one	91.77	coriander, grassy, freshly cut grass
10	938	1071	C_7_H_16_O	4-Methylhexan-1-ol	76.02	grassy, sweet
11	1419	1479	C_9_H_18_O_2_	Decyl acetate	73.15	fresh, fruity, fatty, orange, soapy, waxy
12	2098	2197	C_19_H_38_O	2-Nonadecanone	84.28	**/**
13	1113	1176	C_9_H_18_O_2_	Heptyl acetate	51.72	apricot, fresh, freshly cut grass, pear, rum, woody
14	1442	1479	C_15_H_24_	alpha-Himachalene	90.17	**/**

**Table 3:** Volatile compounds in PPQ were identified using an electronic nose

No.	RI (MXT-5)	RI (MXT-1701)	Molecular formula	Possible volatile compounds	Index of correlation	Sensory descriptions
1	654	740	C_3_H_8_O	3-Methylbutanal	75.38	aldehydic, almond, apple, cheesy, chocolate, fatty, fruity, freshly cut grass, herbal, malty, peach, roasted
2	688	735	C_3_H_8_O	Methyl isobutyrate	78.43	apple, floral, green, fruit, pineapple, sweet
3	952	922	C_10_H_16_	alpha-Pinene	84.36	camphor, orange, earthy, fresh, fruity, freshly cut grass, lime flavor, pine, sweet, terpenic, turpentine, woody

**Fig. 6:**
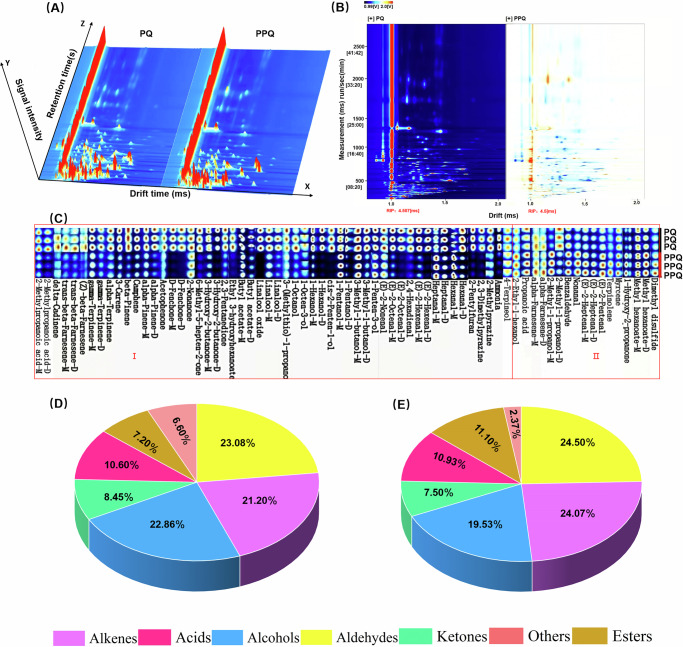
Comparative analysis of volatile components in PQ and PPQ based on HS-GC-IMS. **A** 3D topographic plots of PQ and PPQ. **B** 2D difference plots of PQ and PPQ. Red dots represent compounds with higher content in PPQ samples, blue dots represent those with higher content in PQ samples, and white areas indicate no significant difference between the two groups. **C** Comparison of volatile component contents in PQ and PPQ. Region I represents components with higher content in PQ, while Region II represents those with higher content in PPQ. **D** Proportional distribution of volatile compound classes in PQ. **E** Proportional distribution of volatile compound classes in PPQ.

The 3D plots of PQ and PPQ based on HS-GC-IMS are presented in Fig. 6A. Each peak in the plots represents a volatile compound. The higher the peak, the larger the volume, and the redder the color, the higher the concentration of the compound. To directly compare the differences in volatile components between the two, the spectrum of PQ was subtracted from that of PPQ. This operation yielded a 2D difference plot (Fig. 6B). If the content of volatile compounds was identical, the subtracted background appeared white. The blue color indicated that the peak volume of PQ was higher than that of PPQ, while the red color signified the opposite. Numerous blue regions in Fig. 6B indicate that the content of most volatile components decreased after MHHA. Based on the color variation in the corridor plot shown in Fig. 6C, it was divided into two distinct regions, I and II. Region I represented compounds with higher content in PQ, while Region II represented those with higher content in PPQ. The results indicated that 39 volatile compounds were present at higher levels in PQ (Table 4). These included acetophenone, ethyl 3-hydroxyhexanoate, 3-(methylthio)-1-propanol, delta-cadinene, 4-terpineol, 2-methylpyrazine, 2-pentylfuran, *(Z)*-beta-farnesene, 1-octanol, *(E)*-2-nonenal, *cis*-2-penten-1-ol, 1-octen-3-ol, 6-methyl-5-hepten-2-one, 2,3-dimethylpyrazine, alpha-terpinene, 3-carene, ammonia, and camphene, etc. Among these, components such as 3-(methylthio)-1-propanol, *cis*-2-penten-1-ol, ammonia, and camphene contributed to undesirable odors such as onion, meaty, soy sauce, almond, plastic, and rubber. This demonstrated that MHHA effectively improved the odor quality of PQ by significantly reducing the content of certain undesirable odorants. In contrast, 13 volatile compounds were present at higher levels in PPQ, including propanoic acid, alpha-farnesene, nonanal, *(E)*-2-heptenal, benzaldehyde, methyl hexanoate, myrcene, *(E)*-2-pentenal, 2-methyl-1-propanol, dimethyl disulfide, 2-ethyl-1-hexanol, 1-hydroxy-2-propanone, and terpinolene (Table 4). Among them, 8 compounds including alpha-farnesene, methyl hexanoate, nonanal, myrcene, benzaldehyde, 2-methyl-1-propanol, 2-ethyl-1-hexanol, and terpinolene possessed pleasant aromas such as sweet, grape juice, pineapple, cherry, rosy, woody, lavender, citrus, neroli, and fresh. The content of these favorable aroma substances significantly increased after MHHA. Based on the peak volume data measured for various categories of compounds, a pie chart was plotted as shown in Fig. 6D and Fig. 6E. The proportions of each compound category in PQ and PPQ were calculated separately. In PQ, the proportions were 23.08% (aldehydes), 22.86% (alcohols), 21.20% (alkenes), 10.60% (acids), 8.45% (ketones), 7.20% (esters), and 6.60% (others). The corresponding proportions in PPQ were 24.50%, 24.07%, 19.53%, 10.93%, 7.50%, 11.10%, and 2.37%, respectively. Notably, the proportion of esters with pleasant aromas increased from 7.20% to 11.10%. This change is one of the reasons for the improved aroma of PPQ. The regulation of volatile compounds in PQ by MHHA may be dominated by the HA step. HA could accelerate moisture migration via forced convection, which efficiently removes a large number of undesirable volatile compounds in PQ^30^.

**Table 4:** Volatile compounds were identified from PQ and PPQ samples using HS-GC-IMS

No.	Compound	Formula	Rt(s)	Dt (RIPrel)	Odor characteristics description
1^a^	alpha-Farnesene-M	C_15_H_24_	2730.476	1.43903	citrus, herbal, lavender, neroli
2^a^	alpha-Farnesene-D	C_15_H_24_	2733.951	1.48721	citrus, herbal, lavender, neroli
3^b^	delta-Cadinene	C_15_H_24_	2537.643	1.44041	thyme, herbal, woody
4^b^	Acetophenone	C_8_H_8_O	2489.001	1.17749	sweet, spicy, almond
5^b^	Ethyl 3-hydroxyhexanoate	C_8_H_16_O_3_	2455.993	1.29036	fruity, wood
6^b^	3-(Methylthio)-1-propanol	C_4_H_10_OS	2410.825	1.10040	pungent onion, meaty, soy sauce
7^b^	*trans*-beta-Farnesene-M	C_15_H_24_	2247.525	1.43628	woody, citrus, sweet, green
8^b^	*trans*-beta-Farnesene-D	C_15_H_24_	2245.788	1.49134	woody, citrus, sweet, green
9^b^	*(Z)*-beta-Farnesene	C_15_H_24_	2077.276	1.44178	green, floral
10^b^	4-Terpineol	C_10_H_18_O	1931.349	1.22154	pepper, woody, earthy musty
11^a^	Propanoic acid	C_3_H_6_O_2_	1703.771	1.11692	yogurt, vinegar
12^b^	2-Methylpropanoic acid-M	C_4_H_8_O_2_	1681.187	1.14996	yogurt, rancid cream
13^b^	2-Methylpropanoic acid-D	C_4_H_8_O_2_	1679.450	1.36883	yogurt, rancid cream
14^b^	Linalool-M	C_10_H_18_O	1686.399	1.22291	citrus, rose, woody, blueberry
15^b^	Linalool-D	C_10_H_18_O	1686.399	1.78317	citrus, rose, woody, blueberry
16^b^	1-Octanol	C_8_H_18_O	1656.866	1.47344	citrus, sweet, herbs, waxy, rose, mushroom
17^b^	*(E)*-2-Nonenal	C_9_H_16_O	1494.111	1.41275	fatty, green, waxy, cucumber, melon
18^a^	Benzaldehyde	C_7_H_6_O	1416.541	1.15560	bitter almond, cherry, nutty
19^a^	2-Ethyl-1-hexanol	C_8_H_18_O	1382.926	1.42648	citrus, fresh floral, fatty
20^b^	1-Octen-3-ol	C_8_H_16_O	1221.320	1.16309	mushroom, lavender, rose, hay
21^b^	Linalool oxide	C_10_H_18_O_2_	1139.757	1.25551	floral
22^b^	*(E)*-2-Octenal-M	C_8_H_14_O	1110.127	1.33410	fresh cucumber, fatty, green herbal, banana, green leaf
23^b^	*(E)*-2-Octenal-D	C_8_H_14_O	1112.530	1.81807	fresh cucumber, fatty, green herbal, banana, green leaf
24^b^	D-Fenchone-M	C_10_H_16_O	1061.278	1.29963	mint, camphor, cool
25^b^	D-Fenchone-D	C_10_H_16_O	1062.079	1.78636	mint, camphor, cool
26^a^	Nonanal	C_9_H_18_O	1030.047	1.49267	rose, citrus, strong oily
27^b^	2-Nonanone	C_9_H_18_O	1016.434	1.40856	fresh, sweet, green, herb
28^b^	2,4-Hexadienal	C_6_H_8_O	988.110	1.11242	sweet, green, floral, citrus
29^b^	1-Hexanol-M	C_6_H_14_O	955.814	1.33685	fresh, fruity, wine, sweet, green
30^b^	1-Hexanol-D	C_6_H_14_O	955.045	1.64263	fresh, fruity, wine, sweet, green
31^b^	6-Methyl-5-hepten-2-one	C_8_H_14_O	931.208	1.16713	citrus, fruity, musty, ketonic
32^b^	2,3-Dimethylpyrazine	C_6_H_8_N_2_	918.135	1.11663	nutty
33^b^	*cis*-2-Penten-1-ol	C_5_H_10_O	898.912	1.44205	green, plastic, rubber
34^a^	*(E)*-2-Heptenal-M	C_7_H_12_O	879.688	1.25970	spicy, green vegetables, fresh, fatty
35^a^	*(E)*-2-Heptenal-D	C_7_H_12_O	879.688	1.67069	spicy, green vegetables, fresh, fatty
36^a^	1-Hydroxy-2-propanone	C_3_H_6_O_2_	846.623	1.08016	pungent, caramel, fresh
37^b^	3-Hydroxy-2-butanone-M	C_4_H_8_O_2_	819.183	1.07637	buttery, creamy
38^b^	3-Hydroxy-2-butanone-D	C_4_H_8_O_2_	819.183	1.33142	buttery, creamy
39^b^	Ammonia	NH_3_	780.947	0.85702	ammoniacal
40^a^	Terpinolene	C_10_H_16_	802.259	1.22047	woody, sweet, citrus
41^b^	2-Methylpyrazine	C_5_H_6_N_2_	782.201	1.09039	nutty, musty, roasted, earthy
42^b^	1-Pentanol-M	C_5_H_12_O	763.396	1.25746	balsamic
43^b^	1-Pentanol-D	C_5_H_12_O	763.396	1.51124	balsamic
44^b^	gamma-Terpinene-M	C_10_H_16_	745.846	1.21665	oil, woody, terpenic, lemon, lime, herbal
45^b^	gamma-Terpinene-D	C_10_H_16_	745.846	1.70253	oil, woody, terpenic, lemon, lime, herbal
46^b^	2-Pentylfuran	C_9_H_14_O	731.429	1.25108	beany, fruity, earthy, green, vegetal
47^b^	*(E)*-2-Hexenal-M	C_6_H_10_O	714.505	1.18221	green, banana, fatty
48^b^	*(E)*-2-Hexenal-D	C_6_H_10_O	715.758	1.52144	green, banana, fatty
49^b^	3-Methyl-1-butanol-M	C_5_H_12_O	699.461	1.24598	whisky, banana
50^b^	3-Methyl-1-butanol-D	C_5_H_12_O	698.834	1.49338	whisky, banana
51^b^	Heptanal-M	C_7_H_14_O	664.323	1.35164	fresh, aldehydic, fatty, green herbal, wine, fruity
52^b^	Heptanal-D	C_7_H_14_O	665.282	1.69392	fresh, aldehydic, fatty, green herbal, wine, fruity
53^a^	Methyl hexanoate-M	C_7_H_14_O_2_	651.389	1.26083	pineapple, apricot
54^a^	Methyl hexanoate-D	C_7_H_14_O_2_	650.431	1.68927	pineapple, apricot
55^b^	alpha-Terpinene	C_10_H_16_	647.078	1.21426	woody, terpenic, lemon, citrus
56^b^	1-Penten-3-ol	C_5_H_10_O	626.958	0.94183	ethereal, green, tropical fruits
57^a^	Myrcene	C_10_H_16_	625.520	1.21426	grape juice, spicy, plastic
58^a^	*(E)*-2-Pentenal	C_5_H_8_O	574.741	1.10715	potato, pea
59^b^	3-Carene	C_10_H_16_	539.770	1.21426	citrus, lemon, woody
60^b^	beta-Pinene	C_10_H_16_	539.770	1.21426	resinous, grassy green
61^a^	2-Methyl-1-propanol-M	C_4_H_10_O	500.488	1.17468	fresh, alcoholic, leathery
62^a^	2-Methyl-1-propanol-D	C_4_H_10_O	500.488	1.37143	fresh, alcoholic, leathery
63^b^	Hexanal-M	C_6_H_12_O	485.159	1.27829	fresh, green, fatty, fruity
64^b^	Hexanal-D	C_6_H_12_O	484.680	1.56353	fresh, green, fatty, fruity
65^b^	Butyl acetate-M	C_6_H_12_O_2_	465.997	1.25268	fruity
66^b^	Butyl acetate-D	C_6_H_12_O_2_	466.955	1.61825	fruity
67^a^	Dimethyl disulfide	C_2_H_6_S_2_	461.206	1.15023	sulfurous, cabbage, onion
68^b^	2,3-Pentanedione	C_5_H_8_O_2_	449.709	1.21310	sweet, buttery-creamy, caramel, nutty, cheesy
69^b^	Camphene	C_10_H_16_	435.771	1.20102	woody, camphor
70^b^	alpha-Pinene-M	C_10_H_16_	401.925	1.21556	fresh, camphor, sweet, pine
71^b^	alpha-Pinene-D	C_10_H_16_	402.201	1.66406	fresh, camphor, sweet, pine

M and D represent monomer and dimer of the same compounds, respectively.

^a^Indicates a higher content in PPQ.

^b^Indicates a higher content in PQ.

## Discussion

In this study, we proposed an “HE + H + EH” strategy to investigate the effect of MHHA on PQ by integrating human sensory evaluation (H), electronic tongue (E), HPLC-Q-TOF-MS (H), electronic nose (E), and HS-GC-IMS (H) techniques. Human sensory evaluation and electronic tongue showed that MHHA improved the softness and umami of PQ while reducing its bitterness and earthy flavor. A total of 98 components were identified in PQ and PPQ by HPLC-Q-TOF-MS. MHHA led to a decrease in the content of malonylginsenosides, which was the main reason for the reduction of bitterness in PQ. Simultaneously, MHHA promoted the generation of vinaginsenoside R_7_, ginsenoside Rk_1_, Rg_5_, Rg_6_, and Rh_4_, which demonstrated significant bioactivities beneficial to human health. The combination of electronic nose and HS-GC-IMS enabled the identification of 68 odor compounds in PQ and PPQ. MHHA reduced undesirable odor compounds while increasing pleasant aroma compounds in PQ. These results systematically reveal that the synergistic effects of MH treatment and HA treatment play a crucial role in improving the flavor quality of PQ and promoting the formation of its functional components.

Despite these findings, several limitations still exist. First, the MHHA processing parameters adopted in this study were fixed at 100 °C moist‑heat treatment for 3 h followed by 60 °C hot‑air drying for 10 h. The absence of gradient experiments on parameters prevented us from revealing the dynamic transformation patterns of saponins and volatile compounds during the continuous process. Second, this study only focused on the changes in the chemical composition and sensory flavor of PPQ, and lacked in vivo experiments to further verify the variations in its bioactivity. Third, the current research provided only a short-term static analysis of the finished products after MHHA processing. The lack of long-term storage experiments made it impossible to evaluate the stability of the flavor characteristics and functional components of PPQ during storage. Thus, these limitations indicate directions for subsequent research, including exploring the dynamic changes of components and flavor during MHHA processing, verifying the bioactivity of PPQ through in vivo experiments, and investigating the storage stability of PPQ.

## Methods

### Chemicals and reagents

The ginsenosides Rb_1_, Rb_2_, Rb_3_, and Rc were purchased from Chengdu Must Bio-technology Co., Ltd. 20*(S)*-ginsenoside Rg_3_, 20*(R)*-ginsenoside Rg_3_, and polyphyllin VII were obtained from Chengdu Push Bio-technology Co., Ltd. 20*(S)*-ginsenoside Rg_2_, ginsenosides Rd, Re, and F_2_ were supplied by Chengdu Herbpurify Co., Ltd. The purity of all reference standards is >98%. The C_6_-C_16_ mixed alkanes reference standard was purchased from RESTEK Corporation (USA). Formic acid was purchased from Tianjin Damao Chemical Reagent Factory. Potassium chloride and tartaric acid were purchased from Tianjin Kermel Chemical Reagent Co., Ltd. Purified water was obtained commercially from Hangzhou Wahaha Group. HPLC-grade methanol and acetonitrile were purchased from Oceanpak (Sweden) and Merck (Germany), respectively.

### Preparation of PPQ

Six-year-old PQ samples were collected from Changbai Mountain. The samples were then standardized to ensure uniform size, with lengths of 22.70–24.00 cm and diameters of 1.50–1.70 cm. For the moist-heat treatment, PQ was steamed at 100 °C with a relative humidity of 95% for 3 h. Subsequently, hot-air drying was conducted in an electric thermostatic blast drying oven (Model 101-3BS, Shanghai Lichen Bangxi Instrument Technology Co., Ltd., China). The drying process was carried out at 60 °C with an average internal air velocity of 0.8–1.5 m/s for 10 h until the moisture content stabilized, yielding the final product PPQ. The moisture content was determined by the oven-drying method^31,32^.

### Preparation of test solution

PQ and PPQ were ground separately, passed through a sieve with an aperture size of 355 μm, and prepared for experimental use. For HPLC-Q-TOF-MS analysis, 1.0 g of each type of powder was accurately weighed into a centrifuge tube. Then, 10 mL of 70% methanol was added. The mixture was sonicated at room temperature for 60 min and then centrifuged at 14,000 rpm for 10 min. The supernatant was collected for analysis. In addition, 1.5 g of each powder was accurately weighed separately, mixed with 45 mL of purified water, and ultrasonically treated at room temperature for 30 minutes for electronic tongue analysis. The artificial saliva used in the electronic tongue measurement was prepared by dissolving 2.237 g of potassium chloride and 0.045 g of tartaric acid in 1 L of water.

### Human sensory evaluation

In this study, 57 volunteers (28 males and 29 females, aged 20-25 years) were recruited to conduct human sensory evaluation of sliced PQ and PPQ. All participants had normal taste and olfactory acuity, no habits of smoking or alcohol consumption, and no history of taste disorders. They signed informed consent forms before the experiment, agreeing to the use of their data for academic research and publication. The research process strictly adhered to ethical guidelines, and all acquired data were anonymized. Participants had the right to withdraw from the experiment at any time. Prior to the formal experiment, systematic training was conducted in a sensory analysis laboratory. Theoretical instruction was provided to clarify the definition, perceptual characteristics, and rating criteria of related flavors. Geosmin was used as the reference compound to conduct practical training for earthy flavor recognition^33^. To monitor the actual sensory experience, nose clips were not used during the sensory evaluation process. All evaluations were performed in individual sensory booths to minimize external interference. Sliced samples of PQ and PPQ were placed in disposable odorless trays and presented in coded numerical order using a blind method. The presentation sequence was randomized to avoid order effects. According to the evaluation protocol, participants were required to hold the sample in their mouth and chew for 15 s to ensure sufficient contact between taste receptors and the sample. After each sample, panelists rinsed their mouths thoroughly with purified water and rested for 1 h before testing the next sample to effectively avoid carry-over effects. The samples were evaluated using quantitative descriptive analysis with a 5-point intensity scale for four attributes including earthy flavor, bitterness, softness, and crispness. These four attributes were selected because preliminary experiments indicated that they were significant factors affecting the flavor of PQ, and thus, it was necessary to quantify them further. A score of 0 indicated no perception of the attribute, while a score of 5 represented the maximum perceived intensity. Graphs were generated using Origin 2022 and GraphPad Prism 9.1.0 software^6,34^.

### Electronic tongue analysis

The taste characteristics were evaluated using an SA-402B taste analysis system (Intelligent Sensor Technology Co., Ltd., Japan). The sensor array was composed of bitterness (C00), saltiness (CT0), astringency (AE1), sourness (CA0), and umami (AAE) sensors, along with two reference electrodes. Prior to the experiment, the taste sensors were activated in artificial saliva, and the reference electrodes were activated in a 3.3 M KCl solution. During measurement, each sensor was cleaned in artificial saliva. After the sensors were equilibrated for 30 s, the samples were tested for 30 s. Subsequently, the electronic tongue system converted the collected electronic signals into taste signals. All data were referenced against the artificial saliva^17^.

### HPLC-Q-TOF-MS analysis

An Agilent 1290 HPLC system coupled with a 6550 Q-TOF mass spectrometer was used. The chemical components in PQ and PPQ were analyzed qualitatively and compared in terms of content. Polyphyllin Ⅶ (0.2 mg/mL), which shares structural similarity with ginsenosides, was used as the internal standard. The relative content was derived from the ratio of compound peak area to internal standard peak area. Given that MHHA might significantly affect the content of certain components, compounds with a peak area to internal standard peak area ratio >2 were selected to focus on substances with pronounced changes. A phenyl-hexyl column (4.6 × 50 mm, 3.5 μm) was used for chromatographic separation. The mobile phase consisted of 0.1% formic acid in water (A) and acetonitrile (B). The elution gradient for both PQ and PPQ was as follows: 0–50 min, 5–50% B; 50–55 min, 50–100% B; 55–60 min, 100–100% B. The flow rate was 0.5 mL/min, the column temperature was maintained at 30 °C, and the injection volume was 10 μL. The mass spectrometer parameters were set as follows: gas temperature, 200 °C; gas flow, 11 L/min; nebulizer gas pressure, 35 psig; sheath gas temperature, 350 °C; sheath gas flow, 8 L/min. The fragmentor voltage was 75 V, and the nozzle voltage was 1000 V. The m/z range was set from 100 to 3000. Collision energies were 5, 10, and 15 eV in positive ion mode, and 40, 50, and 60 eV in negative ion mode^35^.

### Electronic nose analysis

Odor analysis of PQ and PPQ was performed using a Heracles Neo ultrafast electronic nose (Alpha MOS, France). The instrument was equipped with a headspace sampler, two flame ionization detectors (FID1 and FID2), and two capillary columns (MXT-5 and MXT-1701; both 10 m × 0.18 mm × 0.4 μm). Briefly, all samples of PQ and PPQ were precisely weighed to 3.0 g and placed in 20 mL headspace vials for 24 h prior to analysis. The acquisition parameters were set as follows: injection volume, 5000 µL; incubation temperature, 80 °C; incubation time, 22 min; injection time, 45 s; initial trap temperature, 20 °C. All other parameters were set to their default values. Furthermore, the temperature program was set as follows: the oven temperature was initially maintained at 50 °C. It was then ramped at a rate of 1.0 °C/s to 80 °C, followed by an increase at 3 °C/s to 250 °C. The final temperature of 250 °C was held for 21 s. A mixture of n-alkanes (C_6_-C_16_) was used as a reference to calibrate retention times. Odor components and their sensory descriptions were identified based on the AroChemBase database^36^.

### HS-GC-IMS analysis

A FlavourSpec HS‑GC‑IMS system (G.A.S., Germany) was employed. The system was coupled with a CTC-PAL3 static headspace autosampler (CTC Analytics AG, Switzerland) and equipped with an MXT-WAX capillary column (Restek, USA). Data processing was performed using the VOCal software. For analysis, 1.0 g of each sample was weighed into a 20 mL headspace vial. After incubation at 80 °C for 20 min, each sample was analyzed in triplicate. The headspace conditions were: incubation at 80 °C for 20 min, a 500 µL injection in splitless mode, an agitator speed of 500 rpm, and a needle temperature of 85 °C. The chromatographic column temperature was maintained at 60 °C. High-purity nitrogen was used as the carrier gas. The injector temperature was set to 80 °C. The ionization source was a tritium ([³H]) source. The drift tube (length: 53 mm) was operated at 45 °C with an applied electric field strength of 500 V/cm. High-purity nitrogen was used as the drift gas at a flow rate of 75 mL/min. The analysis was conducted in positive ion mode. A calibration curve was established using a mixture of six ketone standards, and qualitative identification of the target compounds was achieved by matching to the database of the VOCal software^37^.

### Statistical analysis

Data analysis and visualization were performed using multiple software platforms. Specifically, HPLC-Q-TOF-MS data were processed with Agilent Qualitative Analysis B.06.00 software. The electronic tongue data were modeled by OPLS-DA using SIMCA 14.1 software (Umetrics AB, Sweden) for visual representation of data characteristics. Electronic nose data were analyzed using AlphaSoft V2021 software (Alpha MOS, France) to generate PCA and DFA plots. Radar charts (electronic tongue), fingerprint spectra (electronic nose), and pie charts (HS‑GC‑IMS) were plotted using Origin 2022 software (OriginLab Corporation, USA). Box plots and cluster heat maps were generated with GraphPad Prism 9.1.0 (GraphPad Software, USA). Molecular structure diagrams of the compounds were drawn using ChemDraw Professional 15.1 (PerkinElmer Informatics, USA).

## Supplementary information

Supplementary Information

## Data Availability

The datasets generated and analyzed during the current study are available from the corresponding author upon reasonable request.
