# Supplementary material for: Unveiling the improvement mechanisms of moist-heat and hot-air treatments on the flavor of Panax quinquefolius
Source: NPJ Sci Food. 2026 May 19;10:237. doi: 10.1038/s41538-026-00888-3 (PMC13448238; doi:10.1038/s41538-026-00888-3)
Supplement: Supplementary file 1 [file 41538_2026_888_MOESM1_ESM.docx]

**Unveiling the improvement mechanisms of moist-heat and hot-air treatments on the flavor of *Panax quinquefolius***

Hong-Juan Hou^a^, Wen-Han Pei^b^, Rui-Bo Sun^a^, Chao Wang^c^, Fang-Tong Liu^a^, Tian-Qi Zhang^a^, Gui-Zhong Xin^d^, Cheng Qian^e^, Mu Zhang^f^, Xin-Ru Zhang^a^, Zi-Xuan Ding^a^, Hui Zhang^a^, Tian-Min Wang^a^, Ting-Guo Kang^a^, Hai-Bo Wang^a,∗^, Yue-Hua Chen^a,∗^, Hui-Peng Song^a,∗^

^a^ Key Laboratory for Identification and Quality Evaluation of Traditional Chinese Medicine of Liaoning Province, Liaoning University of Traditional Chinese Medicine, Dalian 116600, China

^b^ The MOE Key Laboratory of Standardization of Chinese Medicines, Shanghai University of Traditional Chinese Medicine, Shanghai 201203, China

^c^ College of Pharmacy, Dalian Medical University, Dalian 116044, China

^d^ State Key Laboratory of Natural Medicines, China Pharmaceutical University, Nanjing 210009, China

^e^ Department of Pathology, School of Basic Medical Sciences, Nanjing Medical University, Nanjing 211166, China

^f^ Department of Endocrinology, Sir Run Run Hospital, Nanjing Medical University, Nanjing 211100, China

* Corresponding authors:

**Hui-Peng Song**

E-mail: songhuipeng15@163.com

**Yue-Hua Chen**

E-mail: chenyuehua527@126.com

**Hai-Bo Wang**

E-mail: [whb_email@126.com](mailto:whb_email@126.com)

**Fig. S1. Total ion chromatograms of PQ (A) and PPQ (B) under positive ion mode.** Peak numbers correspond to saponin compounds listed in Table 1. Chromatographic conditions were consistent across both samples to enable direct component comparison.

**Fig. S2. Discovery of differential components between PQ and PPQ based on relative component content and OPLS‑DA.** (A) OPLS‑DA score plot of PQ and PPQ. (B) Permutation test (n = 200) of the OPLS-DA model. (C) VIP score plot of components in PQ and PPQ. Red indicates VIP > 1.
